# Ibuprofen Compared to Acetazolamide for the Prevention of Acute Mountain Sickness: A Randomized Placebo-Controlled Trial

**DOI:** 10.7759/cureus.55998

**Published:** 2024-03-12

**Authors:** Srinivasa Bhattachar, Vineet K Malhotra, Uday Yanamandra, Surinderpal Singh, Gaurav Sikri, Seema Patrikar, Atul Kotwal

**Affiliations:** 1 Physiology, High Altitude Medical Research Centre, Leh, IND; 2 Space and Environmental Physiology, Institute of Aerospace Medicine, Bengaluru, IND; 3 Internal Medicine, Armed Forces Medical College, Pune, IND; 4 Physiology, Amrita Medical College, Faridabad, IND; 5 Physiology, Armed Forces Medical Services, New Delhi, IND; 6 Community Medicine, Armed Forces Medical College, Pune, IND; 7 Community Medicine, National Health Systems Resource Centre, New Delhi, IND

**Keywords:** incidence, ams, ibuprofen, altitude sickness, acetazolamide

## Abstract

Introduction: Acetazolamide is recommended for the prevention of acute mountain sickness (AMS); however, its use is limited in some areas because of side effects. Previous studies report ibuprofen to be similar to or slightly inferior to acetazolamide. This randomized, triple-blinded, parallel-group, placebo-controlled trial was designed to compare ibuprofen with acetazolamide for the prevention of AMS.

Methods: Four hundred forty-three healthy Asian Indian men with a mean age of 29 (range: 20-49) years were randomized into three groups A, B, and P at 350m (SL). Acetazolamide (A): 85 mg; ibuprofen (B): 600 mg; or placebo (P): calcium carbonate was administered thrice daily, starting one day prior and continuing for three days after arrival at 3500m (HA). Participants were evaluated for AMS using the Lake Louise Questionnaire and for pulse, BP, SpO_2_, and respiratory rate twice daily for the first two days during rest and once a day for days three to six at HA.

Results: Of the 443 participants recruited at SL, 139 could not be airlifted due to logistical limitations, and 304 were available for follow-up at HA. Among these, 254 had ascended as per protocol. By intent to treat (IT) (N = 304; A = 99, B = 102, P = 103), the incidence of AMS (LLQS>/=3) was 12%, 5%, and 13%, and the incidence of severe AMS was 1%, 2%, and 6%, in groups A, B, and P, respectively. Using per protocol analysis (PP) (N = 254; A = 83, B = 87, P = 84), the incidence of AMS was 12%, 6%, and 13% in groups A, B, and P, respectively. The relative risk for developing AMS vs. placebo was A-0.96 (CI:0.46-2.0, p=0.91), B-0.39 (CI:0.14-1.04, p=0.06), A-0.94 (CI:0.42-2.1, p=0.88), and B-0.45 (0.16-1.24, p=0.12) by IT and PP, respectively.

Conclusion: Ibuprofen is effective in males for the prevention of AMS with rapid ascent to 3500 m-rest for the first two days. Acetazolamide was superior to ibuprofen in the prevention of moderate-to-severe AMS.

## Introduction

Lowlanders are at risk of developing acute high-altitude illnesses (HAI) within the first few days of ascent to high altitude (HA>2500m). The risk of HAI, which includes acute mountain sickness (AMS), high-altitude pulmonary edema (HAPE), and high-altitude cerebral edema (HACE), increases with sleeping altitude and rapidity of ascent. AMS is the commonest of all high-altitude illnesses, with an incidence of 25%-49% [1-3]. It is usually benign and self-limiting, but it decreases productivity in the first few days at altitude. Also, untreated, it may, on rare occasions, progress to HACE, especially on further ascent [4].

Although some debate on the exact pathophysiology of AMS remains, AMS is believed to be due to hypoxia-induced intracranial hypertension and vasogenic edema of the brain [5]. This may activate mechanosensitive nociceptive fibers of the trigemino-vascular system [6], leading to headaches and the symptom complex that defines AMS. It has been suggested that free radical-mediated ion pump failure in hypoxia may lead to intracellular edema and that inflammatory mediators may activate the trigeminal vascular system [7]. Thus, inflammation could be a cause for headaches and other symptoms of AMS, which raises the possibility that anti-inflammatory drugs may help prevent AMS. The effectiveness of steroids in the prevention and management of AMS further corroborates the possible role of inflammation [8]. The use of non-steroidal anti-inflammatory drugs (NSAIDs), e.g., ibuprofen, in the treatment of the headache of AMS is advised [8]. Studies comparing ibuprofen to placebo [9,10] found it to be superior to placebo. When compared with acetazolamide, studies found ibuprofen to be similar [11] or slightly inferior [12] to acetazolamide in the prevention of AMS. The HEAT trial showed ibuprofen to be similar to acetazolamide when high-altitude headache was evaluated as the primary objective [11]. A study [12] conducted at White Mountain, California, showed ibuprofen to be slightly inferior to acetazolamide in the prevention of AMS, but the drugs were not compared to placebo. The altitude of recruitment of participants in both trials was above 2500m. The present trial was conducted on healthy lowlander males recruited at near sea level with a uniform ascent profile and passive ascent to high altitude.

## Materials and methods

The study was planned as a randomized, triple-blinded, parallel-group, placebo-controlled trial with an equal allocation of participants, with the primary objective being to compare the effectiveness of ibuprofen, acetazolamide, and placebo in the prevention of AMS. The protocol was approved by the High-Altitude Medical Research Center institutional ethics committee. The study was carried out in the Union Territory of Ladakh, India, between March and October 2015. People ascending from Chandigarh at 350m altitude (SL) by flight to Leh at 3500m (HA) were included in the study. Exclusion criteria were (a) HA native; (b) stay at HA within the last 30 days; (c) history of sulpha drug allergy or intolerance to NSAIDs; (d) history of acid peptic disease; (e) ongoing use of NSAIDS for other complaints; (f) presence of co-morbidities, including acute infections; and (g) history of HAI. Participants were provided printouts detailing the study process and adverse effect profiles of the drugs to be administered. Written informed consent was obtained prior to the onset of data collection in accordance with the Declaration of Helsinki.

To achieve a power of 80% ($\alpha$=0.05, 2-tailed test), assuming a 20% incidence of AMS, to detect an absolute difference of 15%, approximately 80 participants in each group were required to be studied. To cater for attrition, it was planned to study 300 participants, 100 per group, receiving tablets of acetazolamide 85 mg, ibuprofen 600 mg, and calcium carbonate (placebo) 500 mg, respectively, every eight hours. The primary outcome measures of the study were the incidence and severity of AMS, and the secondary outcome measure was the incidence of moderate to severe headache [Lake Louis Score Questionnaire (LLSQ) Headache score>/=2].

Randomization was carried out as per the randomization sequence generated by the trial administrator at High Altitude (HA) using random allocation software version 1.0 developed by the Department of Anaesthesia at the Ishfahan University of Medical Sciences, Ishfahan, Iran. The sequence consisted of 312 participants. The participants were assigned to study groups by research workers based on the sequence generated at SL and were given codes based on the group assigned and the number of participants assigned to the group. Similar-looking tablets for all three arms of the study were manufactured by Radkrish Pharmaceuticals Limited to be dispensed to all participants one day prior to airlift to HA. All medication was placed in number-coded boxes and administered to the participants by research workers at SL. Height and weight were recorded on day three of the stay at HA using a height and weight scale, model M-310800, manufactured by ADE Instruments in Germany. Height was recorded without footwear in an erect posture with the head adjusted in the Frankfurt plane to the nearest 0.5 cm. Weight was recorded in erect posture without footwear to the nearest 0.5 kg. Pulse, BP, respiratory rate, and SpO_2_ were recorded using the Schiller Truscope Classic and Elite models. All participants were made to rest in a supine position for at least 5 minutes before recording vital parameters. SpO_2_ and pulse rate were recorded after an observation period of 30 seconds, and the values appearing for the maximum duration of time were recorded. Blood pressure was recorded using the manual single recording mode of the non-invasive blood pressure module of a multi-parameter monitor in the supine resting position. The respiratory rate was recorded manually after an observation period of one minute.

All data were recorded by workers different from those recruiting the groups and administering the medication. Research workers collecting data, participants in the study, and statisticians analyzing the results were masked with the allocation of participants to the groups. Heart rate, systemic blood pressure (BP), respiratory rate, and arterial HbO2 saturation by pulse oximetry (SpO_2_) were recorded in all participants at SL before the onset of drug prophylaxis. These parameters were again measured, and LLSQ was filled in the morning and evening for the first two days and once daily from days three through six at HA.

Medication was started the day before the ascent to HA by the research worker recruiting participants for the study. Compliance with medication at HA was assessed by research workers different from those recording data or adverse drug reactions. All participants continued to stay at one location for the first six days at HA.

**Figure 1:**
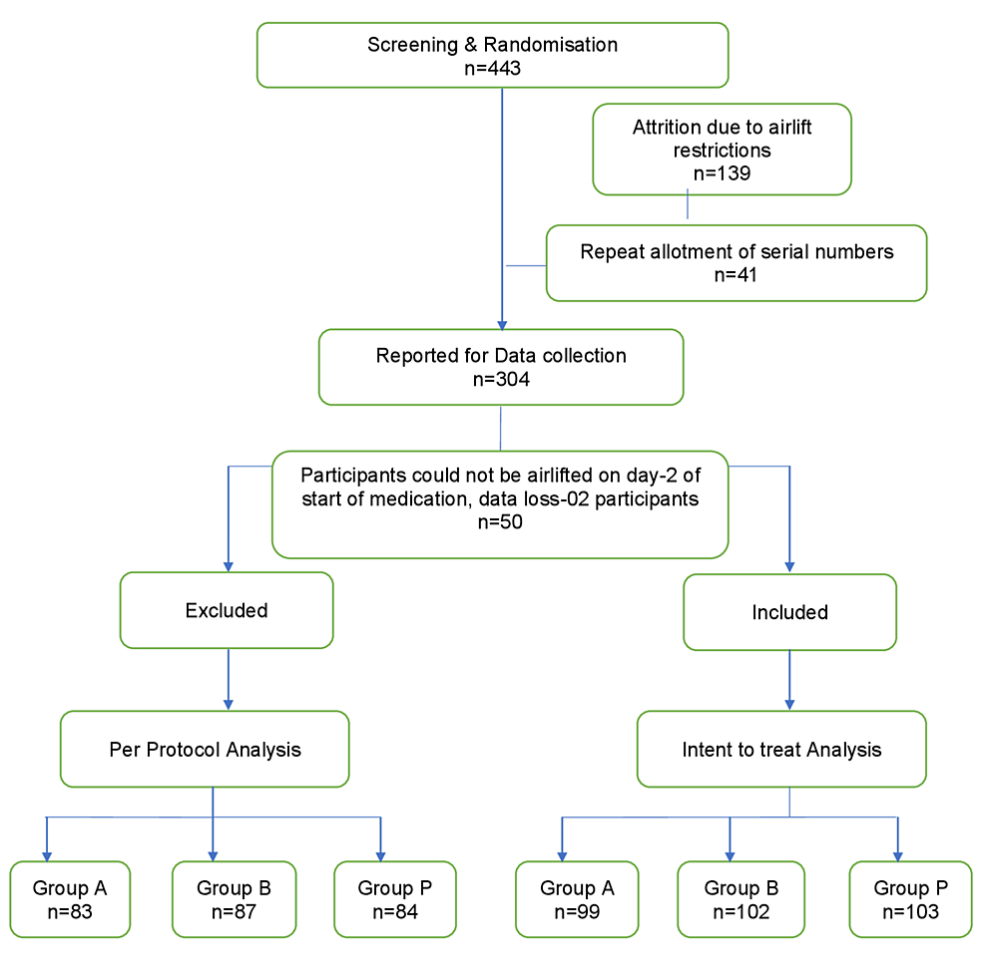
Consort diagram for data collection Groups A: acetazolamide, B: Ibuprofen, P: Placebo

All participants filled out an adverse effects check list twice daily, which was assessed the next morning. Participants were also encouraged to communicate any adverse effects, if any, at any time during the study. Participants reporting adverse drug reactions corresponding to severity (moderate or greater) on a visual analog scale were dropped from the study and managed as per standard management guidelines. All data was entered in digital format and analyzed using IBM Corp. Released 2015, IBM SPSS Statistics for Windows, Version 23.0. Armonk, NY: IBM Corp. Quantitative variables were described using mean (SD) and qualitative variables using percentage. Baseline parameters were compared using a one-way ANOVA for differences in three groups and a t-test for differences in two groups. The incidence and 95% confidence interval were estimated. The difference in incidence rate was tested using the chi-square and exact tests. The trial was registered with DGCTRI, Trial Acknowledgement No. REF/2016/05/011362. The consort diagram is as shown in Figure 1.

Diagnosis of and analysis for AMS

LLSQ was filled by participants on all days of observation. A LLSQ score of ≥3, with headache and at least one more symptom present, was considered diagnostic of AMS. Because AMS was not present in any subject after four days at HA, to factor in the temporal variability in the onset and progression of AMS, the peak LLSQ score in the first four days at HA was considered for comparison between the three groups. A score of ≥5 was considered moderate-to-severe AMS. The study was conducted before the revision of Lake Louise criteria for AMS, which removed the sleep component [13]. Hence, both the sample size calculation and the AMS recorded in the present study are as per previous criteria [14]. The incidence of AMS is presented as per revised criteria for reference. 

## Results

A total of 443 participants were recruited at SL, 304 of whom ascended to HA. Of these, 48 ascended on the third or later day after starting drug prophylaxis and were on medication for two (33), three (7), four (7), and five (1) days before ascent to HA (number of participants shown in parenthesis). Data regarding the date of the start of medication at SL for two participants was not available. The participants were analyzed with the intent to treat. The balance of 254 participants reached HA as per protocol. The trial was stopped after the calculated sample size was achieved. The attrition rate of participants after one day of medication at near sea level due to a delay in flight to HA was 15.78%. Analysis was carried out using both intent-to-treat and protocol techniques. 

The physiological parameters in the three groups are summarized in Table 1.

**Table 1:** Values of physiological parameters in all three groups All values are expressed in Mean (SD). Groups A: Acetazolamide, B: Ibuprofen, P: Placebo, n: number of participants in each group, HR: Heart rate, RR: Respiratory rate, SpO_2_: Saturation percentage of oxygen, Day 1(m): Day 1 morning, Day 1(e): Day 1 evening, Day 2(m): Day 2 morning, Day 2(e): Day 2 evening.

Group		Sea-level	Day 1 (m)	Day 1 (e)	Day 2 (m)	Day 2 (e)	Day 3	Day 4	Day 5	Day 6
A (n=99)	HR (beats/min)	69.49	70.63	73.42	68.69	73.10	71.35	71.00	70.08	71.41
		(9.96)	(10.32)	(12.02)	(10.05)	(11.66)	(12.33)	(11.32)	(10.08)	(10.76)
B (n= 102)		73.18	73.54	75.70	71.41	74.82	73.02	70.81	71.55	71.89
		(11.71)	(12.78)	(12.28)	(12.03)	(11.30)	(13.12)	(11.94)	(12.59)	(11.39)
P (n= 103)		72.11	74.73	78.14	72.93	76.53	74.64	72.26	72.26	72.86
		(10.15)	(12.06)	(11.33)	(11.94)	(11.91)	(11.34)	(12.42)	(11.32)	(10.42)
A	BP-systolic (mm Hg)	118.55	115.08	117.82	117.10	118.02	117.14	116.06	115.82	116.83
		(9.44)	(9.10)	(10.13)	(9.20)	(9.26)	(8.80)	(8.80)	(8.10)	(9.69)
B		118.65	117.30	118.87	120.07	120.55	118.70	116.87	116.45	116.64
		(11.06)	(14.01)	(12.23)	(13.39)	(12.85)	(13.86)	(12.98)	(12.35)	(12.70)
P		118.39	119.49	122.42	123.53	124.17	121.30	120.88	119.88	119.26
		(10.05)	(10.39)	(11.32)	(12.08)	(11.24)	(11.30)	(11.25)	(9.73)	(9.77)
A	BP-diastolic (mm Hg)	68.54	72.16	73.80	73.83	73.45	73.31	72.28	72.86	71.15
		(8.36)	(8.47)	(8.53)	(8.28)	(8.12)	(7.57)	(7.73)	(7.77)	(8.11)
B		69.47	74.72	75.28	75.99	76.91	75.22	73.88	74.27	73.14
		(9.79)	(11.58)	(8.83)	(9.42)	(9.92)	(9.84)	(9.23)	(9.64)	(9.88)
P		69.71	75.29	76.40	77.48	78.13	77.53	76.74	75.59	75.66
		(9.04)	(10.09)	(8.80)	(9.68)	(8.45)	(9.74)	(9.42)	(9.56)	(9.04)
A	RR (per min)	19.15	18.69	19.61	19.83	20.17	20.07	19.78	19.41	19.98
		(1.85)	(2.73)	(2.64)	(3.20)	(3.02)	(3.38)	(3.28)	(3.28)	(2.98)
B		18.97	17.94	19.16	19.76	19.75	19.44	19.79	19.62	19.57
		(1.67)	(3.0)	(3.56)	(4.21)	(3.54)	(3.58)	(3.40)	(3.09)	(3.63)
P		18.39	18.65	19.13	19.20	19.79	19.86	19.56	19.39	19.26
		(1.64)	(3.20)	(3.34)	(3.47)	(3.26)	(3.71)	(3.49)	(3.26)	(3.22)
A	SPO_2 _(%)	96.36	91.06	90.46	92.48	92.15	92.68	94.03	93.56	93.13
		(1.31)	(3.55)	(3.06)	(2.48)	(2.24)	(2.32)	(2.29)	(2.03)	(2.59)
B		96.89	90.90	90.18	91.60	91.45	92.06	92.03	92.20	92.37
		(1.38)	(2.92)	(3.49)	(3.24)	(2.72)	(2.88)	(4.73)	(2.13)	(2.24)
P		96.38	90.76	89.80	91.24	91.07	91.79	91.78	92.29	92.43
		(1.82)	(3.97)	(3.04)	(3.28)	(2.60)	(2.98)	(3.30)	(2.30)	(2.18)

Demographic and vital parameters of the three groups were comparable at SL, except that heart rate and systolic blood pressure values were significantly higher in placebo group compared to other two groups (Table 2).

**Table 2:** Baseline characteristics for all parameters as per intent to treat and per protocol analyses All values are expressed in Mean (SD), n: number of participants in each group, HR: Heart rate, BMI: Body mass index, RR: Respiratory rate, SpO_2_: Saturation percentage of oxygen, $-ANOVA, p<0.05**

Parameter	Intent to treat				Per Protocol Analysis			
	Acetazolamide	Ibuprofen	Placebo	p value^$^(**<0.05)	Acetazolamide	Ibuprofen	Placebo	p value (**<0.05)
n	99	102	103		83	87	84	
Age (years)	29.64	29.55	30.31	0.58	29.14	29.13	30.72	.093
	(5.53)	(5.61)	(6.05)		(5.24)	(5.18)	(5.8)	
BMI	23.67	23.94	23.95	0.71	23.20	23.02	22.68	.785
	(2.91)	(2.56)	(2.58)		(3.8)	(5.11)	(5.72)	
HR (beats/min)	73.42	75.7	78.14	0.02**	69.97	73.26	72.75	.139
	(12.08)	(12.34)	(11.39)		(10.65)	(11.51)	(10.54)	
BP-systolic (mm Hg)	117.82	118.87	122.42	0.01**	119.45	119.67	118.55	.751
	(10.18)	(12.29)	(11.38)		(9.40)	(10.0)	(9.82)	
BP-diastolic (mm Hg)	73.80	75.28	76.40	0.11	68.54	69.55	70.14	.532
	(8.58)	(8.88)	(8.84)		(8.44)	(9.4)	(8.88)	
RR (per min)	19.15	18.97	18.39	0.09	19.23	18.89	18.75	.167
	(1.85)	(1.67)	(1.64)		(1.82)	(1.59)	(1.64)	
SPO_2 _(%)	96.36	96.89	96.38	0.35	96.30	96.80	96.30	.087
	(1.31)	(1.38)	(1.82)		(1.41)	(1.4)	(1.92)	

High altitude illness

Intent to Treat

Incidence of AMS was lowest in ibuprofen group, followed by acetazolamide and placebo groups, as shown in Table 3.

**Table 3:** Incidence and relative risk of AMS in three groups as per intent to treat and per protocol analyses All values expressed in % and number of cases/number of participants in each group, ARR-Absolute risk reduction, 95% CI for relative risk expressed in parenthesis **p<0.05. Participants excluded in the per protocol analysis included two cases of AMS in both the acetazolamide and placebo groups; however, no cases were excluded from the ibuprofen group.

	Intent to treat						Per Protocol					
Group	Incidence in % (no of cases/no of participants in group)	p-value (vs. Placebo)	ARR (vs. Placebo)	Relative Risk (95% CI)	p-value	Incidencein % (no of cases/no of participants in group)		p-value (vs Placebo)	ARR (vs Placebo)	Relative Risk	p-value	
Acetazolamide	12.12%	0.46	0.5	0.96	0.91	12.04 %		0.44	0.75	0.94	0.88	
	(12/99)			(0.46-2.0)		(10/83)				(0.42-2.1)		
Ibuprofen	4.9%	0.03**	7.72	0.39	0.06	5.75 %		0.06	7.04	0.45	0.12	
	(05/102)			(0.14-1.04)		(05/87)				(0.16-1.24)		
Placebo	12.62%					12.79%						
	(13/103)					(11/84)						

The difference in incidence of AMS between the ibuprofen and placebo groups was significant. The risk of developing AMS was lower in the ibuprofen group compared to both the acetazolamide group and the placebo group. The incidence of AMS in the placebo and acetazolamide groups was similar (Table 3).﻿

Per protocol

The demographic and vital parameters of the three groups were comparable at SL (Table 2). The incidence of AMS was lowest in the ibuprofen group, followed by the acetazolamide and placebo groups, though the difference between the groups was not significant. The relative risk for developing AMS was similar to that observed during intent-to-treat analysis (Table 3).

**Figure 2:**
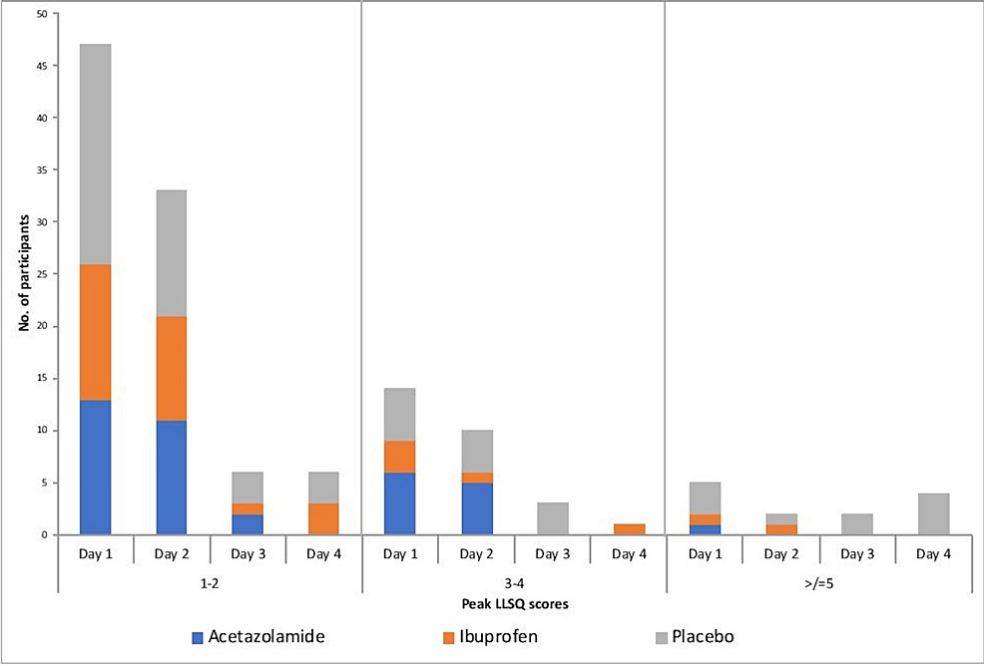
The peak Lake Louis Scores (LLS) of all participants in the three groups have been categorized in three parts LLS 1-2, 3-4 & >/=5 This data has been showed over a period of days 1-4 at HA for temporal representation. (Participants with peak LLSQ score <1 not depicted)

Overall incidence of AMS during the study was 9.93% (CI; 6.92-13.7). The peak LLSQ scores were not different between groups. The peak LLSQ scores of all participants in the groups over four days are depicted in Figure 2.

There was no difference in any of the vital parameters between groups and between participants with AMS or without AMS.

**Figure 3:**
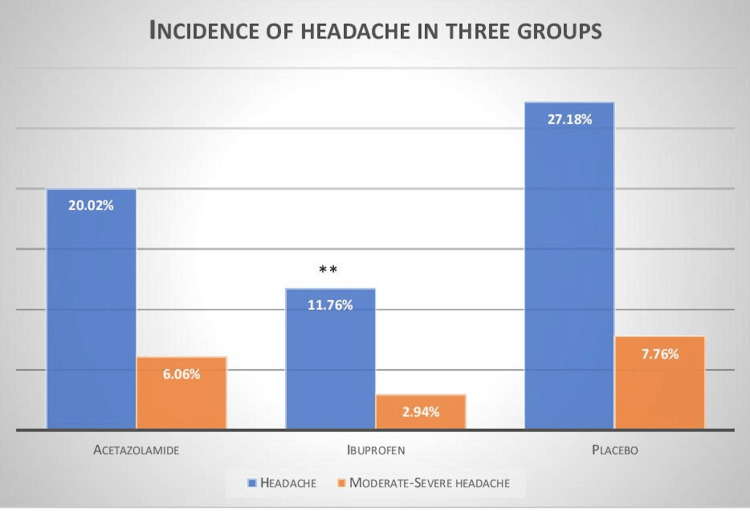
Incidence of headache and moderate-severe headache >/=2 in three groups. (**p=0.003 vs placebo)

In comparison to placebo (5.83% [06/103]) incidence of moderate-severe AMS was significantly lower in acetazolamide group (1.01% [01/99]) p=0.04*), the values in the ibuprofen group were 1.96% [02/102] p=0.09. The incidence of headache was significantly lower in ibuprofen (11.76% [12/102]) compared to placebo group (27.18% [28/103] p=0.003*), as given in Figure 3.

There were no cases of HACE observed during the study. 

Acute Mountain Sickness (AMS) was recorded in the present study as per the Lake Louis Score 1992 [14], with an AMS score of >/=3 considered AMS. Recent network analysis of symptoms shows that sleep disturbance correlates weakly with other symptoms of AMS and is believed to be part of a distinct neurophysiological process in response to hypoxia [15]. In view of these findings, the Lake Louis Score Consensus Committee revised the Lake Louis Symptom Questionnaire (LLSQ), eliminating sleep disturbance as a symptom for the diagnosis of AMS [13] keeping the cut-off LLSQ score as >/=3. In the present study, the findings were recorded as per Lake Louis Score 1992 (as per the process of sample size calculation and the findings of earlier studies utilized in the study design). After eliminating the sleep component of LLSQ, the number of cases of AMS was reduced by 02, 01, and 02 in the acetazolamide, ibuprofen, and placebo groups, respectively, in both intent to treat and per protocol analysis. The findings of the study as per Lake Louis Score 2018 are as depicted in Table 4.﻿

**Table 4:** AMS findings as per Lake Louis 2018 criteria All values expressed in % and number of cases/number of participants in each group, **p<0.05.

	Intent to treat				Per Protocol			
Group	Incidence in % (no of cases)	p-value (vs. Placebo)	Relative Risk	p-value	Incidence in % (no of cases)	p-value (vs. Placebo)	Relative Risk	p-value
Acetazolamide	10.10	0.89	0.95	0.89	9.64	0.89	0.9	0.82
	10/99				08/83			
Ibuprofen	3.92	0.07	0.37	0.08	4.6	0.11	0.43	0.15
	04/100				04/87			
Placebo	10.68				10.71			
	11/103				09/84			

The Chi Square and exact measures of association were not statistically significant. This could be attributed to a further reduction in the incidence of AMS due to the exclusion of cases and sample size limitations, which were calculated based on incidence from studies using LLS 1992. 

**Figure 4:**
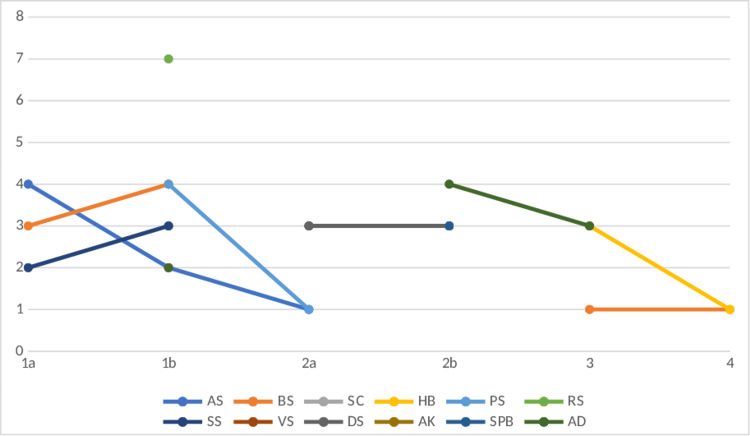
Lake Louis scores of cases AMS in acetazolamide group. Scores represented against the initials of participants during four days after entry at 3500m 1a-day: 1 morning, 1b-day: 1 evening, 2a-day: 2 morning, 2b-day: 2 evening, 3-day: 3, 4-day: 4. Only Lake Louis scores >/=1 shown in the figure.

The participant-wise LLSQ scores are depicted in Figure 4.

**Figure 5:**
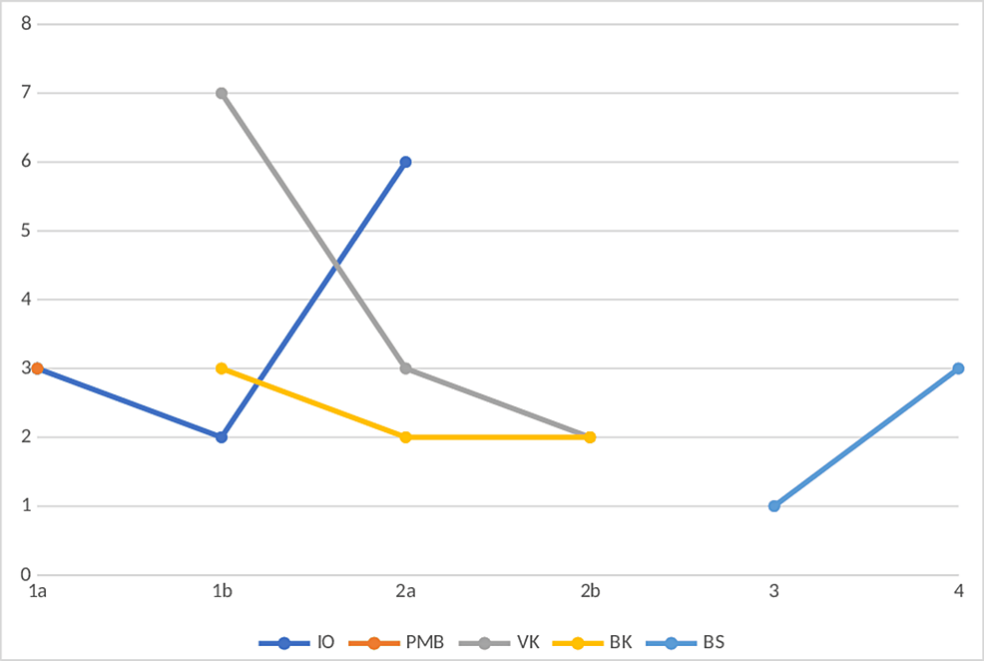
Lake Louis scores of cases of AMS in the ibuprofen group. Scores represent the initials of participants during the four days after entry at 3500m ***Case of AMS with HAPE. 1a-day 1 morning, 1b-day 1 evening, 2a-day 2 morning, 2b-day 2 evening, 3-day 3, 4-day 4. Only Lake Louis scores >/=1 shown in the figure.

**Figure 6:**
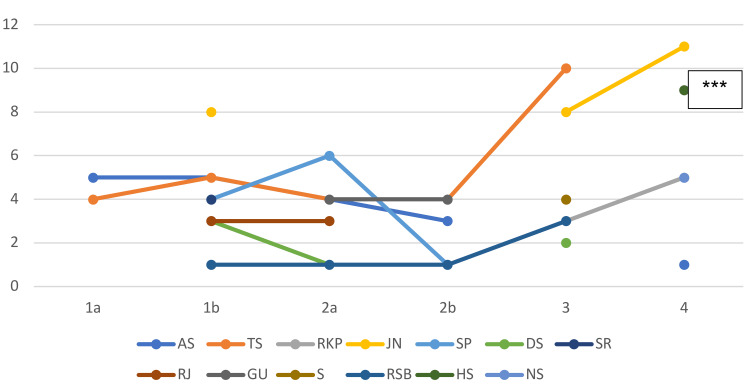
Lake Louis scores of cases of AMS in the placebo group. Scores represent the initials of participants during the four days after entry at 3500m ***Case of AMS with HAPE. 1a-day 1 morning, 1b-day 1 evening, 2a-day 2 morning, 2b-day 2 evening, 3-day 3, 4-day 4. Only Lake Louis scores >/=1 are shown in the figure.

Figure 5 shows the Lake Louis scores of AMS in the ibuprofen group. Figure 6 shows the Lake Louis scores of AMS in the placebo group.

Three study participants suffered from HAPE. One belonged to the placebo group and two to the ibuprofen group, the incidence being 0.97% and 2%, respectively. All three had preceding morbidities. A patient from the ibuprofen group, having developed an upper respiratory tract infection (URTI) the evening before ascent to HA, presented with HAPE on his very first evening at HA. Among others, one (placebo) developed acute URTI after ascent to HA, and the other (ibuprofen) suffered from a tooth infection necessitating extraction. All cases of HAPE recovered uneventfully with treatment.

Adverse effects

The adverse events in both intervention groups were less than placebo, with the ibuprofen group having fewer adverse events compared to the acetazolamide group. Tingling numbness (fingers and toes), dyspepsia, loose stools, and flushing were the most common adverse effects reported by participants in the acetazolamide group, forming 10.26% of all events. Dyspepsia was the most common adverse effect reported by participants in the ibuprofen group, forming 31.82% of all events, as given in Table 5.

**Table 5:** Reported adverse effects of acetazolamide, ibuprofen, and placebo N: Number of participants who reported adverse effects in each group. All values are the number of reported adverse events (%).

	Acetazolamide (n=25)	Ibuprofen (n=20)	Placebo (n=22)
Adverse Effect	Number of events (%)	Number of events (%)	Number of events (%)
Tingling numbness	4 (10.26)	-	-
Burning sensation of stomach/dyspepsia	4 (10.26)	7 (31.82)	11 (24.44)
Loose stools	4 (10.26)	1 (4.55)	5 (11.11)
Feeling of warmth/flushing/burning of body/fever	4 (10.26)	1 (4.55)	1 (2.17)
Pain in abdomen	3 (7.69)	-	
Dryness of throat/nose/mouth	3 (7.69)	1 (4.55)	2 (4.35)
Headache/Heaviness of head	3 (7.69)	3 (13.64)	1 (2.17)
Flatulence	2 (5.13)	-	4 (8.7)
Belching	2 (5.13)	1 (4.55)	4 (8.7)
Increased sleep	2 (5.13)	2 (9.09)	
Fullness of stomach	1 (2.56)	1 (4.55)	10 (21.74)
Constipation	1 (2.56)	-	-
Altered taste in mouth	1 (2.56)	-	1 (2.17)
Throat pain	1 (2.56)	-	-
Increased thirst	1 (2.56)	-	-
Increase in urine	1 (2.56)	-	-
Burning micturition	1 (2.56)	-	
Heaviness & burning of eyes	-	2 (9.09)	-
Bleeding & crusting of nose	-	1 (4.55)	
Dry cough	-	1 (4.55)	
Yellowish discoloration of urine	-	1 (4.55)	
Fever	-	-	1 (2.17)
Urge to pass stools after medication	-	-	1 (2.17)
Total	39	22	46

None of the participants required additional medication or discontinuation of chemo-prophylaxis due to adverse effects. 

## Discussion

The incidence of AMS in the present study was lowest in the ibuprofen group, followed by the acetazolamide and placebo groups. The incidence in the ibuprofen group was significantly lower compared to placebo in the intent-to-treat analysis. The incidence of moderate-severe AMS was significantly lower in the acetazolamide group compared to placebo. 

The incidence of AMS in participants on acetazolamide was similar to that in participants on placebo, with a difference of only one case, less than 1%, between the two groups. Moraga et al. reported similar findings in a study with a similar ascent profile, from near sea level to ~3700m, where six of 12 participants on drug prophylaxis with acetazolamide and seven of 12 participants on placebo developed AMS [16]. No apparent benefit of acetazolamide prophylaxis has been reported in participants ascending by air from sea level to 3200m at the South Pole [17]. The authors of this study state that it was unclear if this lack of effect was due to selection bias. This is in variance with other studies, such as that by Zell et al., in which participants receiving acetazolamide 250mg twice a day had less than half the incidence of AMS of participants on placebo [18]. The degree of reduction in the risk of AMS is associated with the risk of developing AMS in a particular ascent profile [19]; the lower the incidence of AMS, the lesser the effectiveness of acetazolamide. The low incidence of AMS in the present study, as evidenced by the placebo group, could be the reason for the apparent absence of an obvious effect of acetazolamide. The incidence of severe AMS, however, was significantly lower in the acetazolamide group. Also, a reduction in the severity of AMS translates to lesser morbidity, allowing a faster subsequent ascent. The efficiency of acetazolamide prophylaxis in reducing the severity of AMS has been reported earlier [20].

The incidence of AMS in the ibuprofen group was less than half of that in the other groups. The difference was significant for intent-to-treat analysis, probably because of the slightly greater number of participants than in per-protocol analysis. Ibuprofen has been reported to be better than placebo for the prevention of AMS [9,10]. Ibuprofen has been reported to be similar to or slightly inferior to acetazolamide in earlier studies [11,12]. In the earlier study [11], the effects of ibuprofen and acetazolamide on the prevention of AMS were evaluated over an ascent of ~570 to 648m (4280m/4358m to 4928m). This ascent profile would, according to Wilderness Medical Society practice guidelines [8], be assessed as moderate risk for AMS involving physical exertion. Here, ibuprofen was shown to be comparable to acetazolamide and superior to placebo [11]. On the other hand, in a recent study of participants ascending 1240m to 3810m, the incidence of AMS was greater in participants on prophylaxis with ibuprofen than in participants on acetazolamide, although the difference between the groups was less than the pre-specified non-inferiority margin of 26% [12]. Participants in the study traveled from 1240m by vehicle, followed by a 0.8 km hike to 3424 m, and subsequently drove to the staging area at 3545 m, followed by a 4.3km hike to 3810m [12]. This physical exertion could be attributed to the increased risk of AMS and the effectiveness of acetazolamide compared to ibuprofen [21]. Ibuprofen has been shown to be effective in the prevention of AMS compared to placebo [22]. Ibuprofen was marginally better compared to acetaminophen, which has no anti-inflammatory action [23]. Though the exact mechanism is yet to be identified, there is still debate over whether ibuprofen prevents AMS due to its analgesic properties or its anti-inflammatory action [24].

The incidence of AMS in the placebo group in the present study was 12.62% and 12.79% as per intent to treat and per protocol analysis, respectively. This value is lower compared to similar studies carried out at comparable heights [16,25,26]. An incidence of 60% has been reported by a study [25] during passive ascent from 1200m to 3800m. Moraga et al. reported an incidence of 54% in the placebo arm during passive ascent from sea level to 3696m [16]. Kriemler et al. reported an incidence of 38% in males during passive ascent from sea level to 3450/3650m [26]. Participants in the present study took bed rest for the first two days after passive ascent to HA. This could have caused a lower incidence in the present study. Exertion is a known risk factor for AMS [21], and in the study by Kriemler et al., participants ascended on arrival at altitude, which could have contributed to a higher incidence of AMS [26]. Participants in the present study ascended to HA for professional reasons with complete awareness of the implications of HAI. This could have possibly resulted in the early reporting of symptoms, limiting the incidence of AMS, as reported earlier [27]. An incidence of 10% has been recorded during previous work (yet to be published) at the same location. A trend of decreasing AMS incidence is also seen in the studies described above, with recent studies reporting a lesser incidence with comparable ascent profiles [16,25,26]. The sample size limitations of the present, as well as the studies cited above could be another reason for the wide variation in incidence of AMS observed in these studies. 

Thus, from the present study, it appears that ibuprofen 600mg, taken eight hours a day, starting one day before a high-risk ascent to HA, effectively reduces the incidence of AMS in participants who rest for two days after arrival. Participants in the ibuprofen group had fewer adverse events compared to acetazolamide and placebo. Ibuprofen has been reported to have fewer adverse effects compared to placebo earlier [8]. The trials comparing acetazolamide and ibuprofen did not report adverse effects [11,12]. There were two cases of HAPE in the ibuprofen group, one case of HAPE in the placebo group, and none in the acetazolamide group. All cases of HAPE in the study had associated co-morbidities and risk factors like URTI. Though there were participants in the acetazolamide group with similar risk factors, none developed HAPE. This could be attributed to reduced pulmonary vascular resistance, as reported in studies [28,29] among participants taking acetazolamide. In view of the greater number of cases of HAPE in the ibuprofen group and the greater effectiveness of acetazolamide in the prevention of moderate-severe AMS, based on the present evidence, ibuprofen is not to be preferred over acetazolamide for the prevention of AMS.

Limitations of the study

The participants of the present study were healthy Indian males between the ages of 20 and 45. The limited sex and age of participants limit the wider extrapolation of the findings. Restriction of the study to a single location, in ascenders with exactly the same ascent profile, limits extrapolation of the findings of the study at greater heights and with different modes and/or patterns of ascent. The non-standard dose of acetazolamide used for the purpose of masking is not commercially available or practical for prophylaxis. The effect of ibuprofen in the prevention of AMS could be attributed to its effect on the prevention of headaches. This effect cannot be discerned from the prevention of AMS in the present study and could be addressed in future studies. The cases of severe AMS in the study were limited to unequivocally stating that one intervention arm was better than the other. 

## Conclusions

Acetazolamide is the recommended drug for the pharmaco-prophylaxis of AMS, with a wide array of physiological effects that support the use of this drug for the prevention of AMS. Ibuprofen given at 600 mg thrice a day provides better pharmaco-prophylaxis for AMS on ascent by air to 3500m and rest for the first two days in healthy Asian Indian males compared to placebo. It may be considered an alternative in participants requiring prophylaxis but in whom acetazolamide is contraindicated or not well tolerated.
